# An 18-year-old female with recurrent esophageal variceal bleeding

**DOI:** 10.4103/0256-4947.51811

**Published:** 2009

**Authors:** Omar J. Shah, Parveen Shah, Irfan Robbani, Farooq Mir, Parvez Nazir

**Affiliations:** a From the Department of Surgery, Sher-i-Kashmir Institute of Medical Sciences, Kashmir, India; b From the Department of Pathology, Sher-i-Kashmir Institute of Medical Sciences, Kashmir, India; c From the Department of Radiodiagnosis, Sher-i-Kashmir Institute of Medical Sciences, Kashmir, India

An 18-year-old female with recurrent esophageal variceal bleeding due to extrahepatic portal venous obstruction was referred to us for surgical management. On general physical examination she was pale. There was no jaundice or lymphadenopathy. Abdominal examination disclosed a massive splenomegaly (12 cm below the costal margin). Laboratory tests revealed a hemoglobin of 85 g/L, a total leucocyte count of 4.2×10^9^/L and the platelets were 30×10^9^/L. Liver function tests and other biochemical investigations were within normal limits. Color Doppler sonography revealed multiple collateral channels replacing the portal vein (portal cavernoma) and massive splenomegaly. Furthermore, it demonstrated multiple, small foci of echogenic nodules with no acoustic shadowing in the spleen. Non-enhanced CT divulged massive splenomagaly with a pressure effect on the ipsilateral kidney and multiple, discrete, millimetric hyperdense spots in the spleen (Figure 1). A contrast-enhanced splenoportovenogram confirmed the presence of portal cavernoma, esophageal varices, multiple perisplenic collaterals and extensive thrombosis of the splenoportal venous axis. A diagnosis of extrahepatic portal vein obstruction leading to portal hypertension was made.

**Figure 1:**
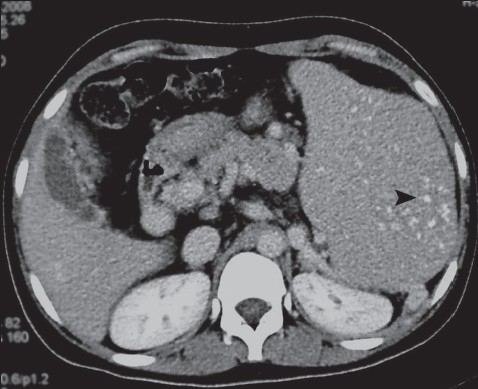
Non-enhanced abdominal CT scan showing massive splenomegaly with multiple tiny, discrete, hyperdense lesions within the spleen (black arrow).

Pneumococcal and *Haemophilus influenzae* vaccines were given and the patient underwent a modified Suguira procedure consisting of extensive esophagogastric devascularization combined with esophageal transaction, reanastomosis and splenectomy. The postoperative hospital period was uneventful and the patient was discharged on the tenth postoperative day. Histopathological examination of the spleen disclosed an abnormality.

1. What is the preoperative diagnosis of the splenic lesions?
2. What is the differential diagnosis of small, discrete, hyperdense splenic lesions?

## Gamna-Gandy bodies

This patient had ultrasound evidence of splenomegaly with discrete, multiple, small echogenic foci in the splenic parenchyma. Both color Doppler and CT splenoportovenography revealed evidence of extrahepatic portal hypertension. In addition, CT confirmed the presence of multiple hyperdense foci in the splenic parenchyma (Figure 1). In this clinical setting, the splenic lesions represent Gamna-Gandy bodies (GGB).

The differential diagnosis of small, discrete, hyperdense splenic lesions apart from GGB would also include sarcoidosis, miliary tuberculosis, histoplasmosis and *Pneumocystitis carinii* infection.

**Figure 2:**
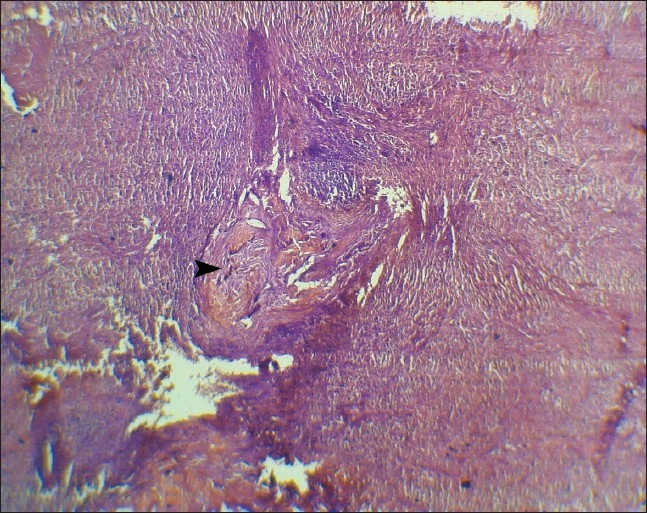
Photomicrograph of spleen showing darkly stained Gamna-Gandy bodies (arrow) in a background of granulomatous vasculitis (hematoxylin-eosin, ×100).

**Figure 3:**
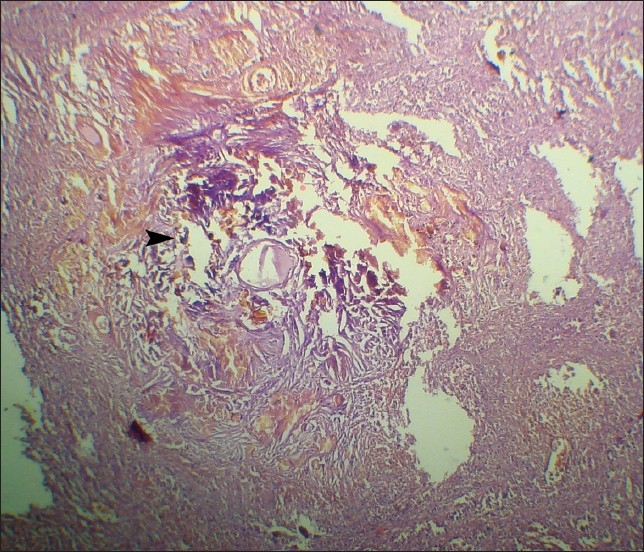
Photomicrograph showing granulomatous vasculitis, sclerosis, hyalinization and fragmentation of elastic fibers with fibroblastic reaction and darkly stained spheroid Gamna-Gandy bodies (arrow) (hematoxylin-eosin, ×100).

Microscopic examination of the splenic lesions revealed typical spheroid, bamboo-shaped fibers resembling mycelial structures (Figure 2, 3). There was also evidence of infiltration of the macrophages and foreign body giant cells. Hence, a histopathological diagnosis of GGB in the spleen was made.

## DISCUSSION

Gamna-Gandy bodies (GGB), also known as siderotic nodules or tobacco flecks, are minute, firm nodules of fibrous tissue impregnated with iron pigments (hemosiderin) and calcium salts. They occur as a result of focal hemorrhages and necrosis followed by accumulation of hemosiderin. The most common cause of GGB in the spleen is portal hypertension; it is observed in 9% to 12% of these patients.^1^,^2^ They have been also seen in conditions like paroxysmal nocturnal hemoglobinum uria,^3^ hemolytic anemia, sickle cell anemia, leukemia,^4^ lymphoma,^5^ angiosarcoma,^6^ in patients receiving blood transfusions and in acquired hemosiderosis.^7^ However, it is unclear whether in such conditions their occurence is a result of primary disease or associated portal hypertension.

Rarely GGB may be seen in extrasplenic sites like in cardiac myxoma,^8^ renal cell carcinoma,^9^ ovary,^10^ liver,^11^ thymoma,^12^ follicular adenoma of thyroid,^13^ retroperitoneal lymph nodes 12 and in central and peripheral nervous system neoplasms.^14^ Although Tedeschi et al^13^ described the various histological features of GGB, the presence of spheroid bamboo-shaped or articulated fibers that resemble mycelial structures are considered pathognomic of GGB.

In portal hypertension these lesions should be distinguished from sclerotic venous branches within the spleen, which shows the presence of blood flow within them on Doppler imaging. On sonography, multiple hyperechoic foci within the spleen may also be seen in sarcoidosis,^15^ histoplasmoisis, tuberculosis,^16^ and disseminated *Pneumocystitis carinii* infection.^17^ Such conditions can be excluded by proper clinical history and other classical features. A non-enhanced CT scan detects GGB as multiple faint high attenutation spots within the spleen; however, their detection depends on the amount of calcium deposition. MRI is described as the most sensitive method for detection of GGB. Due to their hemosiderin content, such lesions are characterized by low signal intensities on all pulse sequences. The gradient echo sequence is considered to be highly sensitive for detection of hemosiderin and typically shows the blooming effect.^18^

Thus in patients of portal hypertension, detection of such lesions on various imaging modalities not only strengthens the diagnosis of portal hypertension, but also excludes other causes like miliary tuberculosis, histoplasmosis and disseminated *Pneumocystitis carinii* infections. This report also provides a correlation of various imaging modalities with histopathological diagnosis.
